# Maternal C3 complement and C-reactive protein and pregnancy and fetal outcomes: A secondary analysis of the PEARS RCT-An mHealth-supported, lifestyle intervention among pregnant women with overweight and obesity

**DOI:** 10.1016/j.cyto.2021.155748

**Published:** 2021-10-23

**Authors:** Maria A. Kennelly, Sarah Louise Killeen, Catherine M. Phillips, Gouiri Alberdi, Karen L. Lindsay, John Mehegan, Martina Cronin, Fionnuala M. McAuliffe

**Affiliations:** a UCD Perinatal Research Centre, School of Medicine, University College Dublin, National Maternity Hospital, Dublin 2, Ireland; b School of Public Health, Physiotherapy and Sports Science, University College Dublin, Dublin 4, Ireland; c Department of Pediatrics, University of California, Irvine, Irvine, CA, USA; d Susan Samueli Integrative Health Institute, UCI College of Health Sciences, Irvine, CA, USA; e National Maternity Hospital, Dublin 2, Ireland

**Keywords:** Complement component 3, C-reactive protein, Overweight, Obese, Pregnancy, Lifestyle intervention

## Abstract

**Objectives:**

Elevated circulating levels of complement component 3 (C3) and C-reactive protein (CRP) have been linked with adverse pregnancy outcomes. Lifestyle interventions may hold potential to ameliorate these effects. We investigated the effect of an antenatal healthy lifestyle intervention on maternal C3 and CRP concentrations and assessed their relationship with maternal and fetal metabolic markers and outcomes.

**Study design:**

Secondary analysis of data from the **P**regnancy **E**xercise **A**nd **N**utrition **R**esearch **S**tudy (PEARS) randomized controlled trial.

**Methods:**

Women (n = 406) with C3 and CRP concentrations determined in early pregnancy (14–16 weeks) and/or late pregnancy (28-weeks) with corresponding fasting glucose, insulin, c-peptide, and lipid profiles were included in the analysis. Pregnancy outcomes included: diagnoses of gestational diabetes (GDM), pre-eclampsia (PET) or pregnancy induced hypertension (PIH), pre-term birth (delivery < 37 weeks), low birth weight (<2500 g), small-for-gestational age (SGA) defined using < 5th or 10th centile for birthweight and cord blood measures of glucose and lipid metabolism. T-tests investigated changes in C3 and CRP over time. Chi-square, Pearson’s’ correlations and multiple regression investigated relationships with outcomes.

**Results:**

The PEARS intervention did not influence maternal C3 or CRP concentrations in pregnancy. There was no relationship between CRP concentrations and any maternal or infant outcome. Women who developed GDM had higher C3 concentrations in early (*p* = 0.01) and late pregnancy (*p* = 0.02). Women who developed PIH/PET had lower C3 concentrations in early (*p* = 0.02), but not late (*p* = 0.10) pregnancy. Maternal C3 concentrations in early pregnancy were a small but significant predictor of maternal insulin concentrations in early (β = 0.40, 95% CI 0.27, 0.53; *p* < 0.001) and late (β = 0.30, 95% CI 0.17, 0.43*p* < 0.001) pregnancy, early total cholesterol (TC), and both early and late triglycerides, LDL and HDL Cholesterol concentrations (all *p* < 0.001). Women who delivered SGA babies (<10th centile) had lower C3 concentrations than women who did not in both early (*p* < 0.001) and late pregnancy (*p* = 0.01). No relationship between maternal C3 or CRP and fetal glucose concentrations or lipid profiles was observed.

**Conclusion:**

Maternal C3 may play a role in multiple adverse pregnancy outcomes including cardiometabolic ill-health. Further research on this, and strategies to reduce C3 in a pregnant population, are warranted.

## 1. Introduction

Heightened systemic inflammation plays a considerable role in the development of adult cardiometabolic diseases such as insulin resistance, altered lipid metabolism, atherosclerosis and hypertension [1]. Outside of pregnancy, elevated C3 complement concentrations (C3) are a risk factor for the metabolic syndrome (MetS), a condition characterised by dyslipidemia, elevated arterial blood pressure, abdominal obesity, and disordered glucose regulation [2,3]. Pregnancy is an intricate physiological process during which remarkable transformations occur in the cardiovascular and immune systems of the mother to ensure a successful pregnancy and birth [4]. The complement system plays an essential role in the innate immune activation and response, and in placental and fetal development during pregnancy [4,5]. As a result, concentrations of complement components may change naturally throughout a healthy pregnancy with a gradual incline in complement C3 [6,7]. Disorders of complement regulation have been linked with pregnancy complications including miscarriage, growth disorders and medical complications in the mother such as hypertension and gestational diabetes [4]. Higher concentrations of maternal C3 complement component (C3) and C-reactive protein (CRP) have been associated with obstetric outcomes such as preterm birth [8].

Diet and lifestyle factors may influence C3 concentrations [9]. Physical activity could have a role in attenuating complement activation as evidenced by lower C3 levels in marathon runners compared to sedentary controls [10]. A cross-sectional study of adults reported that reallocating 30 min of sedentary time with moderate to vigorous physical activity was associated with a more favourable inflammatory profile including lower C3 concentrations [11]. Dietary fat intake and smoking may also modulate the relationship between elevated circulating C3 concentrations and MetS risk, further highlighting the potential role for healthy lifestyle interventions to optimise inflammatory status [12]. CRP, an acute phase reactant, has been independently associated with cardiovascular events in the general adult population [13]. In pregnancy, elevated CRP has been linked with hypertensive diseases and fetal growth restriction [14]. Similar to C3, diet and lifestyle factors may also influence CRP concentrations [15,16]. A multi-disciplinary programme aimed at reducing weight through lifestyle changes in pre-menopausal women with obesity reported a reduction of markers of inflammation including CRP [17]. Similarly in a young adult female population, a low glycemic index (GI) diet has also been shown to attenuate circulating CRP levels [18].

Diet and exercise advice are integral to the management of pregnant women [19]. In pregnancy, limited evidence from randomised controlled trials suggests that a healthy lifestyle package including a low-glycemic diet and physical activity advice, can reduce CRP [20,21]. To the best of our knowledge however, there is no data on the impact of a healthy lifestyle package on maternal C3 concentrations. Therefore, our objective was to examine the effect of the Pregnancy Exercise and nutrition Research Study (PEARS) lifestyle intervention (a eucaloric, low GI diet and 30 min of daily moderate physical activity) on CRP and C3 concentrations in a pregnant population. We also investigated associations of CRP and C3 with a range of pregnancy and birth outcomes including gestational diabetes (GDM), pre-eclampsia (PE), pregnancy induced hypertension (PIH), pre-term delivery, low birth weight, small-for-gestational age (SGA) defined using < 5th or 10th centile for birth-weight, and markers of fetal lipid and glucose metabolism.

## 2. Methods

### 2.1. Study design

**Fig. 1.**
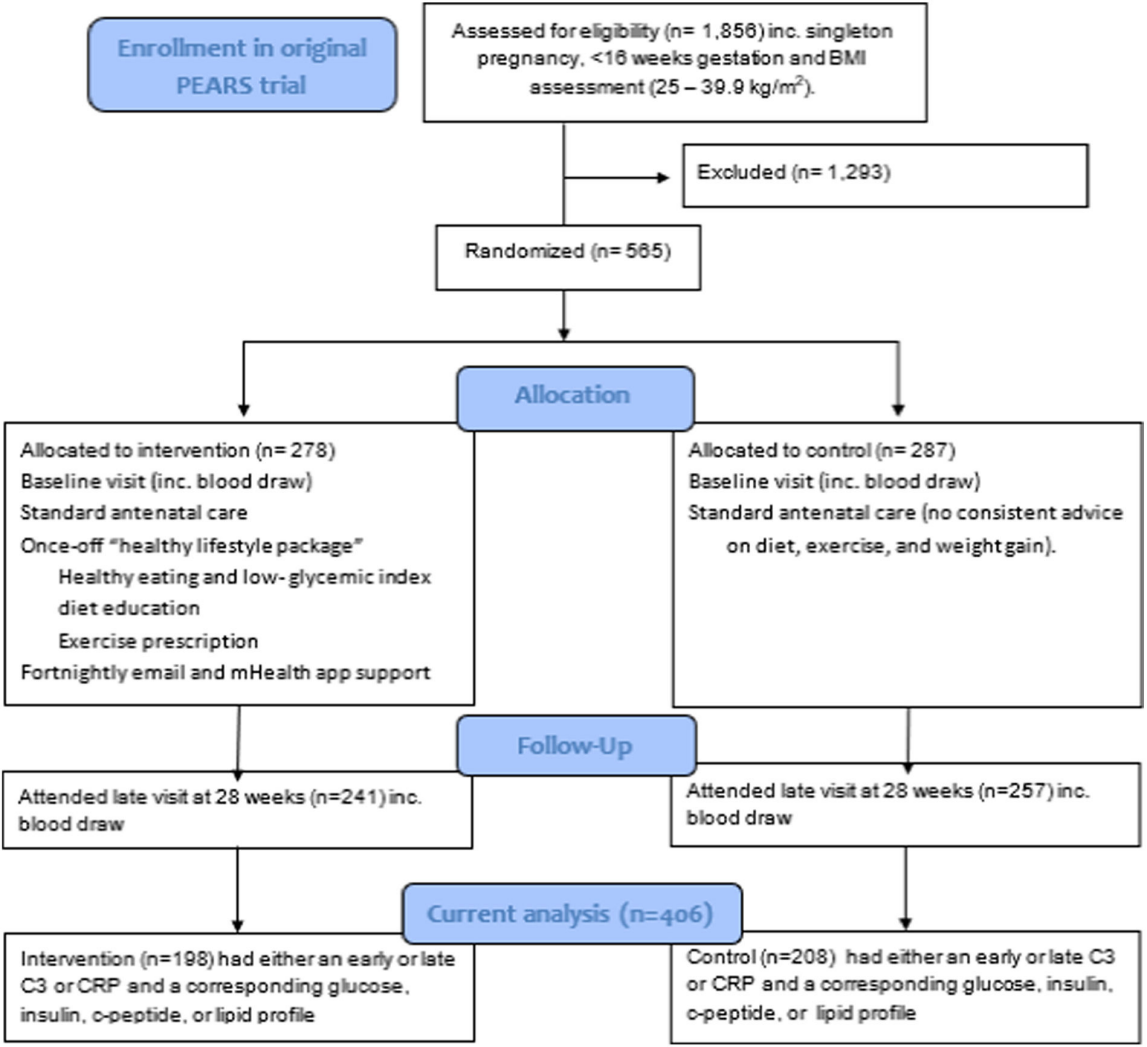
Flow chart of PEARS trial and subject selection for the current analysis, adapted from Kennelly et al. 2018 (24).

This is a secondary analysis of participants recruited as part of the Pregnancy Exercise And nutrition Research Study (PEARS) study. The PEARS study (ISRCTN registry, https://www.isrctn.com/, ISRCTN29316280) was conducted between March 2013 and August 2016 with institutional ethical approval from the National Maternity Hospital and written maternal consent. This was a randomised controlled trial of a mobile health (M—Health) behavioural lifestyle intervention with smartphone app support to prevent GDM in an overweight and obese pregnant population. Details of the study protocol and cost analysis have been published previously [22,23]. In brief, the intervention consisted of a once off education session at study entry which included low GI eucaloric dietary advice provided by a research dietitian or nutritionist, and an exercise prescription of 30 min of physical activity for five days a week, provided by an obstetrician. This was re-enforced through a specifically designed smart-phone app, fortnightly emails and two face-to-face study visits, all underpinned by behaviour change theory. The primary outcome of the trial was the incidence of GDM diagnosed per the International Association of Diabetes in Pregnancy Study Groups criteria at 28–30 weeks’ gestation [22,24]. While the intervention had no effect on the diagnosis of GDM, it resulted in lower GI food intakes and greater exercise participation [17]. Further details of the trial results have been published elsewhere [25]. In this analysis, we use data collected as secondary outcomes in the PEARS trial to explore the potential effect of the PEARS intervention on maternal C3 and CRP, and relationships between maternal C3 or CRP concentrations and maternal and fetal outcomes (Fig. 1).

### 2.2. Study sample

Women were screened for eligibility for the study at their first antenatal visit through a review of their medical chart. Women were eligible if they had a BMI of between 25.0–39.9 kg/m^2^, singleton pregnancy and absence of previous GDM or any other medical illness requiring treatment. The PEARS trial involved pregnant women (n = 565) with overweight or obesity and the primary outcome data was incidence of diabetes determined with oral glucose tolerance test (OGTT) values. For the current analysis, 406 women with sufficient serum sample to allow for determination of C3 and CRP concentrations in early (14–16 weeks) and/or late (28-weeks) pregnancy, and measurement of the corresponding fasting glucose, insulin, c-peptide, or lipid profiles were included. Each of these markers were secondary outcomes of the trial and were subject to missing data in the case of insufficient serum samples or inability to attend study visits, outside of the primary outcome data collection.

### 2.3. Data collection

All women had their height and weight measured by a qualified professional in early pregnancy (baseline visit, approximately two weeks post their first antenatal visit) and late pregnancy (28-week follow up). Additional demographic information including maternal age, ethnicity and smoking status were collected in early pregnancy. Fasting (minimum 8 h) blood samples were collected from the mothers in the morning in early and late (28 weeks) pregnancy. Cord bloods were collected at delivery [22]. Diet was assessed using three-day food diaries in early (14–16 weeks gestation - preintervention) and late pregnancy (28 weeks gestation – post intervention) [22]. The glycemic index and glycemic load of foods were calculated from food diaries analyzed with Nutritics Professional 3.09 [24].

### 2.4. Biochemical analyses

Plasma glucose was analyzed following centrifugation by hospital laboratory staff at the shortest possible interval following sample collection using the AU680 Chemistry analyser (Beckman Coulter Inc., High Wycomb, UK) and the hexokinase method. Bloods collected for serum analysis of insulin, lipid and inflammatory markers were centrifuged at 4 °C within an hour of sample collection for 5 min. The separated serum was immediately frozen at −20 °C, with subsequent transfer to a −80 °C freezer. Insulin and c-peptide were quantified by automated immune-assay (Roche Cobas 602; Roche Diagnostics, Basel, Switzerland) with typical CVs < 5%. Total cholesterol, high density lipoprotein cholesterol (HDL-C) and triglycerides were analyzed on a Roche Cobas 702 analyser (Roche Diagnostics). Low density lipoprotein cholesterol (LDL-C) was estimated using the equation of Friedewald et al. [26]. Maternal C3 was analyzed according to the immunoturbidimetric assay for serum complement C3 (Rx Daytona; Randox Laboratories, Antrim, UK). CRP was analyzed using a biochip array (Evidence Investigator™ Metabolic Syndrome Array II Randox Laboratories, Antrim, UK).

### 2.5. Maternal and fetal outcomes

The primary outcome of this study was the effect of the PEARS lifestyle intervention on circulating levels of C3 and CRP, two markers of maternal inflammation. The secondary outcomes of this analysis relates to maternal and infant health; including incidence of maternal GDM diagnosed per the International Association of Diabetes in Pregnancy Study Groups criteria at 28–30 weeks’ gestation [22], PET or PIH, markers of maternal and fetal glucose and lipid metabolism (insulin, LDL-C, HDL-C, triglycerides, total cholesterol) and birth outcomes including pre-term delivery (delivery < 37 completed weeks), small for gestational age (SGA, birthweight < 10th or 5th centiles) and low birth weight (birth weight < 2500 g) [27,28].

### 2.6. Covariates

The analysis is controlled for known confounders of inflammation including maternal age (years), ethnicity (Caucasian/non-Caucasian), BMI (weight/height^2^) and smoking status in early pregnancy (yes/no).

### 2.7. Statistical analysis

All statistical analyses were performed using IBM SPSS software for Windows version 22.0 (SPSS Inc, Chicago, IL). Data was assessed for normality and skewed data were log transformed prior to analysis. This included maternal CRP, insulin, c-peptide, gestational age, BMI, early triglycerides, and all cord markers except for C3. Independent sample t-tests were used to compare normally distributed continuous data between the intervention and control groups. Paired sample t-tests using data split by study group (intervention or control) were used to compare continuous data within each group. To compare categorical data between the intervention and control group, χ^2^ tests were used. Relationships between metabolic variables and inflammatory markers were assessed using Pearson correlations. The variables that were significantly correlated with maternal C3 and CRP concentrations were further analyzed using multivariate regression analysis and the standardised Beta coefficients reported. We adjusted for a range of confounders including maternal age, ethnicity, BMI (obesity or overweight), parity, GDM diagnosis and smoking status in early pregnancy. Given the exploratory nature of the study, we did not control for study group. In additional analyses, we ran both the primary and secondary outcome separately based on BMI category (greater than and<30 kg/m^2^) to investigate for the impact of BMI on these outcomes.

## 3. Results

Details of baseline maternal and fetal demographics are presented in Table 1. In brief, mean age and BMI of the participants was 32.49 ± 4.35 years and 29.24 ± 3.35 kg/m^2^ in the intervention and control groups, respectively. This was a predominantly Caucasian population (90.6%). The women with available C3, CRP and cardiometabolic marker data for analysis were distributed evenly between the intervention (n = 198) and the control (n = 208) groups. The women with missing data who were not included in this analysis (n = 158), were also distributed evenly between intervention (n = 81) and control (n = 78) groups. There were no differences in glycemic index, glycemic load or exercise between intervention and control groups at baseline (Table 1), In late pregnancy, the intervention group had significantly lower glycemic index, glycemic load and higher self-reported exercise compared to the control group (Table 1).

### 3.1. Effect of the PEARS intervention on maternal inflammation

No differences in maternal C3 and CRP concentrations were observed between intervention and control groups. Both groups experienced increased C3 concentrations from early to late pregnancy (Table 2) however the mean change in C3 from baseline to 28 weeks (15.4 ± 18.2 mg/dl for the intervention group and 17.0 ± 21.6 for the control group) was not different between groups. In paired sample t test, there was no statistically significant difference in concentrations of maternal CRP from early and late pregnant in the intervention (*p* = 0.50) and control group (*p* = 0.09). In the separate analyses for overweight and obesity, there was no difference in either C3 or CRP between intervention or control groups (all *p* > 0.05).

### 3.2. Maternal inflammation and maternal pregnancy outcomes

Women with GDM had higher circulating C3 in both early and late pregnancy, compared to women without GDM at each timepoint (Table 3). Women diagnosed with PET and PIH had lower C3 in early pregnancy only. There were no statistically significant differences in CRP between women with and without GDM, PET or PIH, at any time-point. In the sub-analysis based on BMI, the difference in C3 in women based on diagnosis of GDM was seen in those with obesity (180.1 ± 24.05 vs 166.8 ± 26.25, p = 0.04) but not overweight (158.5 ± 27.7 vs 150.9 ± 24.32 mg/dl, p = 0.118). The difference in C3 based on PET or PIH was seen in those with overweight (135.7 ± 25.8 vs 152.2 ± 24.6 mg/dl, p = 0.04) but not obesity (154.2 ± 24.11 vs 170.23 ± 25.57 mg/dl, p = 0.116)

### 3.3. Maternal inflammation and maternal metabolic profiles

Correlation analysis revealed multiple positive, significant albeit weak correlations between early pregnancy C3 concentrations and early cardiometabolic markers including total cholesterol (Pearson correlation *r* = 0.312, *p* < 0.001), LDL cholesterol (*r* = 0.330, *p* < 0.001), triglycerides (*r* = 0.438, *p* < 0.001) and c-peptide (*r* = 0.160, *p* = 0.004). There was a weak significant negative correlation between early pregnancy C3 and early HDL cholesterol (*r* = −0.225, *p* < 0.001). Early pregnancy C3 was correlated with late pregnancy insulin (*r* = 0.357, *p* < 0.001). Table 4 outlines several other weak positive correlations between early pregnancy C3 and other late cardiometabolic markers including total cholesterol, HDL cholesterol and LDL cholesterol (all correlation coefficients below 0.3 and p values < 0.05). Regarding C3 concentrations in late pregnancy, they were positively correlated with late maternal insulin (*r* = 0.334, *p* < 0.001). There were additional weak but significant relationships with c-peptide, total cholesterol, LDL cholesterol and triglycerides (all correlation coefficients below 0.3 and p values < 0.05). There was a negative correlation with late HDL (*r* = −0.267, *p* < 0.001). There were no significant correlations between log transformed CRP in early or late pregnancy and maternal cardiometabolic markers at any timepoint, except for a weak correlation with triglycerides (Table 4).

In the multiple linear regression model, adjusted for age, BMI category, smoking status, GDM diagnosis, parity, and ethnicity, maternal C3 concentration in early pregnancy was a significant predictor of insulin concentrations in early pregnancy (β = 0.40, 95% CI 0.27, 0.53; *p* < 0.001). For each unit increase in maternal C3 concentrations (mg/dl), maternal insulin concentrations increase by 0.44 mmol/L. This relationship persisted in late pregnancy with early maternal C3 concentrations predicting late maternal insulin (mmol/L) (β = 0.21, 95% CI 0.09, 0.32*p* < 0.001). Early maternal C3 concentration also was a significant predictor of total cholesterol (mmol/L) in early pregnancy (β = 0.34, 95% CI 0.06, 0.11*p* < 0.001), and suggestive of a relationship in late pregnancy *p* = 0.05. Early C3 was associated with early (β = 0.44, 95% CI 0.44, 0.66*p* < 0.001) and late (β = 0.30, 95% CI 0.17, 0.43*p* < 0.001) triglyceride concentrations; early (β = −0.16, 95% CI −0.27, −0.05*p* < 0.001) and late HDL cholesterol in mmol/L (β = −0.25, −0.06 95% CI −0.39, −0.01*p* < 0.001) and early (β = 0.34, 95% CI 0.32, 0.47*p* < 0.001) and late (β = 0.25,95% CI 0.12, 0.35*p* < 0.001) LDL cholesterol. The associations between early pregnancy CRP and early triglyceride were not significant in the adjusted multiple linear regression model (*p* = 0.583). The relationship with cord HDL was no longer significant after controlling for confounders.

### 3.4. Birth outcomes and fetal metabolic profiles

There were no significant differences in early and late pregnancy C3 or CRP concentrations between mothers who did and did not experience a pre-term birth, although the data suggest a potential relationship for early C3 concentrations (*p* = 0.06) (Table 5). When stratified based on BMI above or below 30 kg/m^2^, lower C3 in early pregnancy was seen in women with obesity who experienced preterm birth (146.9 (4.6) vs 169.8 (26.3) mg/dl, p < 0.001) but not in those with overweight (152.2 (25.0) vs 150.6 (21.5) mg/dl, *p* = 0.83). Looking at birth weight above or below 2500 g, there were no significant differences in either C3 or CRP concentrations at any timepoint (all p values > 0.05). When taking gestational age into account however, there were significant difference between groups. Women who gave birth to SGA infants (birthweight < 10th centile), had lower C3 concentrations in early and late pregnancy compared to women who gave birth to infants without growth restriction (*p* value < 0.05). When stratified by BMI above or 30 kg/m^2^, a lower C3 in women with an SGA < 10th centile infant was seen only in women with overweight (134.9 (29.6) vs 153.5 (23.6) mg/dl, *p* = 0.003) but not in those with obesity (148.2 (27.1) vs 168.9 (26.3) mg/dl, *p* = 1.83). There were only 3 women with obesity who delivered an SGA < 10th centile infant compared to 16 women with overweight. When SGA was defined by birthweight < 5th centile, differences were only observed with early pregnancy C3 concentrations. (Table 4). Table 5 illustrates that there were no differences noted in early or late pregnancy CRP concentrations regarding SGA using either the 5th or 10th centile (all *p* values > 0.05).

## 4. Discussion

Our study generates new data on maternal C3 during pregnancy and to our knowledge, this is the first examination of a pregnancy intervention on C3 concentrations and associations with both maternal and fetal outcomes. The PEARs healthy lifestyle intervention appears to have had no effect on maternal C3 and CRP levels. When looking at the individual markers, there was no relationship between CRP and any maternal or fetal outcome. Importantly, at a metabolic level, a significant relationship was observed between maternal C3 concentrations and markers of maternal insulin resistance and lipid metabolism. In addition, women with GDM had higher C3 concentrations than women without this diagnosis.

Our findings of a null effect of the PEARS antenatal lifestyle intervention are consistent with a secondary analysis of the multicentre LIMIT study which revealed that a complex behavioural intervention delivered antenatally in women with overweight and obesity at baseline had no effect on markers of inflammation including CRP, tissue necrosis factor-alpha (TNF-α) and interferon gamma [29]. A smaller pregnancy intervention study (n = 382) by McCarthy et al., involving targeted weighing and dietary advice, also yielded similar results with respect to CRP [30]. He et al. used data from women with a healthy BMI and found that levels of C3 rise gradually throughout pregnancy from a mean of 93.5 mg/dL in the first trimester, to 102.8 mg/L at 13–19 weeks and 119.9 mg/dL at 36 weeks [6]. The mean early-pregnancy concentrations of C3 in our study participants were higher than those found by He et al. at 156.9 mg/dl and 159.8 mg/dl in the intervention and control group respectively. All women in our study had a BMI over 25 kg/m^2^. Levels of C3 have been shown to rise with increasing BMI [31] and are associated with body fat percentage, even in those with a healthy BMI [32]. Evidence in men suggests that abdominal adipose tissue may have high expression of genes for C3 in those with obesity [33].

Targeting C3 reduction may be a useful route to slow down the progression of cardiometabolic disease [34]. In non-pregnant adult populations, a loss of>10% of overall body weight may be required to see concomitant changes in inflammatory markers including CRP [35]. A previous study found that that a very low-calorie diet (603 kcal/day) for six weeks could reduce C3 levels from baseline in individuals with obesity as it was associated with over 10% weight loss [9]. This level of weight loss is not appropriate for a pregnant population. During pregnancy, incremental weight gain according to pre-pregnancy BMI is expected to support fetal development and tissue deposition [36]. The women who participated in the PEARS study were therefore advised to consume a eucaloric low GI diet. It is possible that although the degree of gestational weight gain was limited with the PEARS intervention, the weight gain experienced throughout pregnancy attenuated the potential impact of the lifestyle intervention on C3 and CRP concentrations.

Moderate to vigorous physical activity is associated with a more favourable inflammatory profile [11]. A study examining the influence of theoretical replacement of sedentary time with physical activity on inflammatory markers revealed a more favourable inflammatory profile, characterized by higher adiponectin and lower C3, leptin, interleukin 6 and white blood cell concentrations, when 30 min sedentary time was substituted by an equivalent amount of moderate to vigorous physical activity but not light activity [37]. In the PEARS study, the intervention group saw a significant increase in their moderate intensity exercise by approximately 18 min a week while the control group saw no difference over the study period. The percentage of women in the intervention group meeting the American College of Obstetrics and Gynaecology exercise prescription, of 30 min moderate intensity physical activity at least five times a week, was low (21.6%) and this did not significantly change after the trial [25]. It is possible therefore that although the intervention group saw significant improvement in their physical activity levels, the overall duration of activity undertaken per week was insufficient to elicit a change in inflammatory markers. Diet and lifestyle interventions may benefit from targeted behavioural change components that address specific issues likely to have the greatest effect on desired outcomes, such as inflammation [38]. Future trials that involve physical activity as part of a lifestyle intervention should therefore consider emphasizing and evaluating the type and intensity of exercise as well as the optimal duration to achieve a favourable change in inflammation.

The complement system contributes to immune function and inflammation through cross-talk with immune cells and interaction with systems such as coagulation [34]. Uncontrolled or excessive activation may therefore contribute to inflammatory disease development or progression [34,39]. A review article by Copenhaver et al. suggested that increased C3 levels are directly linked to independent MetS components, including triglycerides and lipids in non-pregnant populations [40]. Associations between C3 gene polymorphisms and MetS risk have been reported [41,42]. In addition, high levels of C3 may be a marker and risk factor for diabetes [43,44]. In this exploratory analysis, maternal C3 levels had significant, positive correlations with maternal markers of insulin and lipid metabolism and multiple regression analysis suggested predictive value. In both early and late pregnancy, women with GDM had higher C3 compared to those with normal glucose tolerance. When we stratified the analysis based on BMI however, we observed the relationship between C3 and GDM only in those with obesity. The relationship between obesity, inflammation and GDM should therefore be considered when interpreting these findings.

The complement system is necessary for a symbiotic maternal-fetal-placental relationship. A pathological complement activation may lead to significant adverse outcomes that can affect the fetus and mother in the short and longer term [4]. In this exploratory work, women delivering SGA or PET/PIH had lower C3 concentrations compared to those who did not experience these complications. Additionally, when we stratified results by BMI, lower C3 was found in women with obesity who experience preterm birth, compared to those who delivered after 37 weeks. Complement system activation earlier in pregnancy has been found to predict pre-term birth in some studies [45-47]. In addition, there is some evidence to suggest that complement C3 has a role in fetal growth, although this is not universal in the literature [48,49]. Some studies have found no differences with respect to C3 concentrations between women with and without PET/PIH while others suggest increased C3 may increase risk of PET [49-52]. Soto et al. reported lower C3 in both SGA and PET/PIH however their findings did not reach statistical significance [49]. In animal studies, complement activation, particularly C5a, is associated with angiogenic factor imbalances that are associated with placental dysfunction [53]. Although speculative it is possible that complement C5a and other proteins were activated in the women experiencing placental dysfunction in this study, resulting in lower C3 compared to those who did not. While we did not measure the full suite of complement proteins, the significance of these complications on maternal and fetal health and the novelty of the findings support further research on complement proteins in pregnancy.

Overall, our results suggest that obesity during pregnancy may predispose to higher inflammation and GDM while lower pre-pregnancy BMI may be associated with more complex inflammatory changes to the complement system that may play a role placental function during pregnancy. A limitation of our study was that it was not possible to provide data on fetal C3 concentrations due to the haemolysed nature of the cord samples obtained. Strengths of our study includes our extensive examination of a range of maternal biomarkers of glucose homeostasis, inflammation, and lipid profiles in a well characterised at-risk pregnant cohort during early and late pregnancy (data available on > 70% participants at both time points) and the inclusion of fetal cardiometabolic markers and birth outcomes. This data enables us to investigate the complex interplay between inflammatory markers and biomarkers of cardiometabolic health. Also, while a growing body of evidence on the effects of lifestyle interventions in pregnancy on CRP exists, this is one of few studies examining the influence of a healthy lifestyle intervention on maternal C3 concentrations and its associations with pregnancy and birth outcomes. The ethnic breakdown of the women in our study is reflective of the population in Ireland however this was a post-hoc analysis of a single centre RCT with a predominantly third level educated population with a higher socioeconomic status [24]. The results of this secondary analysis, therefore, should be interpreted with caution, although they serve to support hypothesis generation for future studies

## 5. Conclusion

This study is an exploratory analysis of the relationship between maternal C3 and CRP, a lifestyle intervention, and adverse pregnancy, birth, and metabolic outcomes. While the PEARS lifestyle intervention did not influence C3 or CRP concentrations, our findings demonstrate further novel links between C3 Complement protein and maternal and birth outcomes. Lower maternal C3 concentrations were found in women who experienced pre-term delivery, SGA and PET/PIH. In GDM, levels of C3 were higher however obesity may be a factor in the relationship between C3 and glucose tolerance. In addition, maternal C3 was associated with maternal insulin resistance and unfavourable lipid profile, and multiple regression analysis suggested a relationship with cardiometabolic health. Together, these findings present novel data describing the potential role of C3 in pregnancy outcomes and warrant further research.

## Figures and Tables

**Table 1:** Baseline Characteristics of study population.

Baseline characteristics	n = 406	Intervention (n = 198)	Control (n = 208)	*p* value
Maternal age, mean years (SD)	32.5 (4.4)	32.9 (4.6)	32.2 (4.1)	0.107
Maternal BMI, median kg/m^2^ (IQR)	28.3 (26.7, 32.2)	28.3 (26.7, 31.5)	28.1 (26.6, 31.2)	.423^a^
Gestational age at delivery, mean days (SD)	283 (275, 288)	283.0 (275.0, 288.0)	283.5 (276.0, 289.0)	0.218
Birth weight, mean grams (SD)	3650.3 (548.0)	3609.0 (540.0)	3688.7 (551.7)	0.170
Primiparous n (%)	205 (51.6)	95 (49.2)	110 (53.9)	0.349
White n (%)	368 (90.6)	184 (96.4)	184 (90.2)	0.016
Smokers n (%)	19 (5.4)	8 (4.6)	11 (6.2)	0.503
Incidence GDM n (%)	53 (13.9)	27 (14.6)	26 (13.2)	0.693
Early glycemic index mean (SD)	58.7 (4.9)	58.9 (4.9)	58.8 (5.0)	0.349
Early glycemic load median (IQR)	132.9 (111.0, 154.4)	131.7 (109.2, 153.5)	135.7 (112.1, 156.3)	0.362
Early exercise Mets median (IQR)	396.0 (99.0, 727.5)	396.0 (99.0, 699.0)	396.0 (99.0, 735.0)	0.665
Late glycemic index mean (SD)	57.3 (57.6)	56.5 (4.6)	58.0 (4.8)	0.008
Late glycemic load median (IQR)	121.8 (99.27, 147.8)	111.1 (96.6, 129.4)	131.3 (105.7, 155.8)	<0.001
Late exercise Mets median (IQR)	518.9 (198.0, 720.0)	537.0 (297.0, 840.0)	480.0 (159.0, 693.0)	0.005

Data presented as mean (SD), median (IQR) or N (%). BMI, Body Mass Index. SD = Standard deviation. IQ = interquartile range. Data include both intervention and control group. MET = metabolic equivalent of task. *p* values are comparing intervention and control groups. The *p* values for continuous variables were determined through independent sample t-tests and Pearson chi-square statistics for categorical variables.

a = log transformed data used.

**Table 2:** The effect of the PEARS lifestyle intervention on C3 and CRP.

C3	Intervention		Control		*P* value
C3 Early (mg/dl)	174	156.9 (25.5)	179	157.8 (27.0)	0.78
C3 Late (mg/dl)	173	172.4 (26.4)	188	175.5 (27.4)	0.27
		*p < 0.001		*p < 0.001	
Mean change C3	150	15.4 (18.2)	158	17.0 (21.6)	0.49
**CRP**					
CRP Early (mg/L)^a^	168	4.2 (11.8)	167	2.6 (3.8)	0.61
CRP Late (mg/L)^a^	163	3.4 (7.09)	179	2.7 (2.9)	0.64
		*p = 0.49		*p = 0.09	
Mean change CRP^a^	139	−0.6 (11.9)	147	−0.7 (5.1)	0.65

Data presented as mean (standard deviation). *p* values derived from independent sample t-tests.

* *p* value derived from paired sample t tests.

a denotes log transformed data. C3, complement component 3; CRP- c reactive protein. Early refers to baseline bloods taken between 14 and 16 weeks and late refers to bloods taken at 28 weeks’ gestation.

**Table 3:** C3 and CRP concentrations according to pregnancy complications.

C3 (mg/dl)	GDM				*P*	PET or PIH				*P*
	Diagnosed		Not diagnosed			Diagnosed		Not diagnosed		
	n	Mean (SD)	n	Mean (SD)		n	Mean (SD)	n	Mean (SD)	
Early	49	167.0 (28.2)	280	156.0 (25.7)	0.01	17	141.8 (25.1)	293	157.7 (26.2)	0.02
Late	49	184.5 (33.7)	303	172.7 (25.4)	0.02	20	164.3 (24.9)	298	174.8 (27.6)	0.10
Mean change C3 CRP (mg/L)	45	16.8 (18.2)	254	16.3 (20.5)	0.87	16	16.7 (17.1)	259	16.1 (20.7)	0.90
Early^a^	47	6.11 (16.2)	265	3.06 (7.1)	0.61^a^	16	3.7 (7.6)	279	3.5 (9.5)	0.93^a^
Late^a^	47	4.1 (10.0)	288	2.89 (4.2)	0.52^a^	18	3.6 (3.5)	283	3.0 (5.8)	0.18^a^
Mean change CRP	43	0.1 (12.8)	236	−0.4 (8.4)	0.74	14	−0.8 (7.8)00	241	−0.3 (9.6)	0.82

* *P* value derived from independent sample t-tests.

a denotes p value generated using log transformed data. C3, complement component 3; CRP- c reactive protein. GDM, gestational diabetes; PET, Pre-eclampsia; PIH, pregnancy induced hypertension. Early refers to baseline bloods taken between 14 and 16 weeks and late refers to bloods taken at 28 weeks’ gestation. Data from both the intervention and control group are used.

**Table 4:** Associations between maternal C3 and CRP with maternal and cord biomarkers.

	Early pregnancy						Late pregnancy					
	C3			CRP^a^			C3			CRP^a^		
	*n*	*r*	*p*	*N*	*r*	*p*	*n*	*r*	*p*	*n*	*r*	*p*
Early pregnancy												
Glucose	311	0.196	0.001	293	−0.075	0.202		–			–	
Insulin^a^	338	0.494	<0.001	320	−0.039	0.484		–			–	
C-peptide^a^	330	0.106	0.053	312	−0.037	0.512		–			–	
Total cholesterol	338	0.312	<0.001	320	−0.001	0.988		–			–	
Triglycerides^a^	338	0.447	<0.001	320	−0.160	0.004		–			–	
HDL Cholesterol	338	−0.225	<0.001	320	0.025	0.660		–			–	
LDL Cholesterol	338	0.330	<0.001	320	0.027	0.627		–			–	
Late pregnancy												
Insulin^a^	305	0.357	<0.001	298	0.033	0.572	338	0.334	<0.001	319	−0.041	0.461
C-peptide^a^	304	0.088	0.124	297	0.049	0.404	338	0.125	0.022	319	−0.052	0.352
Total cholesterol	304	0.150	0.009	298	−0.003	0.959	336	0.226	<0.001	317	−0.059	0.296
Triglycerides	304	0.269	<0.001	298	−0.119	0.041	336	0.258	<0.001	317	0.004	0.949
HDL Cholesterol	304	0.266	<0.001	298	0.066	0.259	336	−0.194	<0.001	317	0.003	0.959
LDL Cholesterol^a^	304	0.201	<0.001	298	0.019	0.743	336	0.246	<0.001	317	−0.054	0.338
Cord blood												
Insulin^a^	168	0.028	0.719	159	0.068	0.394	186	0.039	0.599	181	−0.029	0.699
C-peptide^a^	173	0.093	0.223	164	0.077	0.318	192	−0.028	0.699	186	−0.096	0.935
Total Cholesterol^a^	169	0.077	0.318	159	−0.041	0.606	190	−0.021	0.770	183	0.067	0.366
Triglyceride^a^	169	−0.047	0.546	159	0.059	0.461	190	−0.126	0.083	183	0.061	0.414
HDLCholesterol^a^	169	0.198	0.010	159	−0.091	0.256	190	0.065	0.376	183	0.054	0.468
LDLCholesterol^a^	169	0.026	0.742	159	−0.003	0.966	190	−0.025	0.734	183	0.055	0.458

HDL, high density lipoprotein; LDL, low density lipoprotein.

a Denotes log transformed. Early refers to baseline bloods taken between 14 and 16 weeks and late refers to bloods taken at 28 weeks’ gestation. Data for this table comes from both the intervention and the control group.

**Table 5:** Maternal C3 and CRP concentrations in early and late pregnancy and birth outcome.

C3 (mg/dl)	Gestational age (weeks)				*p*	SGA (BW< <10th centile)				*p*	SGA (BW < 5th centile)				*p*
	< 37/40		>37/40			SGA		Not SGA			SGA		Not SGA		
	n	Mean (SD)	n	Mean (SD)		n	Mean (SD)	n	Mean (SD)		n	Mean (SD)	n	Mean (SD)	
Early	17	148.7 (18.4)	324	158.0 (26.7)	0.06	19	137.0 (28.9)	307	158.7 (25.6)	<0.01	9	138.5 (36.9)	317	158.0 (25.8)	0.03
Late	14	169.9 (21.8)	337	174.4 (27.1)	0.54	20	160.3 (33.7)	318	175.8 (26.3)	0.01	9	159.2 (41.3)	329	175.3 (26.3)	0.05
Mean change C3 CRP (mg/L)	13	18.5 (20.2)	286	16.1 (20.2)	0.68	16	21.6 (20.3)	270	16.6 (20.2)	0.34	7	22.3 (24.0)	279	16.7 (20.2)	0.47
Early^a^	17	2.1 (2.3)	308	3.5 (9.2)	0.80^b^	18	2.3 (1.9)	293	3.3 (7.6)	0.62	9	2.2 (1.6)	302	3.4 (7.7)	0.98
Late^a^	12	3.3 (3.0)	321	3.0 (5.5)	0.19^b^	18	1.6 (1.6)	302	3.0 (5.2)	0.41	7	−0.1 (0.7)	313	3.1 (5.3)	0.52
Mean change CRP	11	1.1 (3.5)	268	−0.4 (9.3)	0.62	14	−0.8 (2.2)	252	−0.3 (9.6)	0.86	6	−9 (1.5)	260	−0.3 (9.5)	0.88

*P* values calculated by independent sample t-tests.

a denotes log transformed data. C3, complement factor 3; CRP, c- reactive protein. BW birthweight; SGA small for gestational age.
